# LTSS IS LOCAL, EVEN IN COUNTRIES WITH NATIONAL LTSS PROGRAMS

**DOI:** 10.1093/geroni/igz038.564

**Published:** 2019-11-08

**Authors:** Pamela Nadash

**Affiliations:** University of Massachusetts Boston, Boston, Massachusetts, United States

## Abstract

This study reports the results of a cross-national qualitative assessment of how different countries structure their provision of long term services and supports (LTSS). It emphasizes the universality of the local role, even in countries that offer some form of universal coverage for LTSS. At minimum, countries devolve the responsibility for administration and eligibility determination to sub-national units, variously called provinces, départements, Länder, or other terms. However, many countries do much more than that: subnational units can be responsible for the safety net welfare programs that pick up the costs that the universal programs do not cover. They may also run other programs that affect the ability of people with LTSS needs to live good lives, such as housing and health programs; again, the role of sub-national governments often focuses on those least able to pay. In addition, in some countries, local governments have a role in helping to finance the national program as well. Differing abilities to support these responsibilities across regions can result in geographic disparities in access to care – so, too, can differences in administration and eligibility determination, resulting in many of the same issues that we in the US confront regarding access to LTSS through the Medicaid program. Thus, even countries with strong national programs for LTSS experience many of the same tensions between national and sub-national units of government that we in the US do.

